# The Interface Thermal Resistance Evolution between Carbide-Bonded Graphene Coating and Polymer in Rapid Molding for Microlens Array

**DOI:** 10.3390/polym13142334

**Published:** 2021-07-16

**Authors:** Xiaohua Liu, Cheng Guo, Yandong Liu, Feng Wang, Yanfeng Feng

**Affiliations:** 1 College of Mechatronics and Control Engineering, Shenzhen University, Shenzhen 518060, China; liuxh89@126.com (X.L.); cheng.guo@szu.edu.cn (C.G.); 2 College of Physics and Optoelectronic Engineering, Shenzhen University, Shenzhen 518060, China; 3 College of Mechanical Electrical and Engineering, Hebei Normal University of Science & Technology, Qinhuangdao 066004, China; liuyd65@126.com (Y.L.); wangfengwwff@126.com (F.W.); 4 School of Sino-Germany Intelligent Production, Shenzhen Institute of Technology, Shenzhen 518116, China

**Keywords:** polymer optics, surface rapid heating, interface thermal resistance, FEM simulation

## Abstract

Surface rapid heating process is an efficient and green method for large-volume production of polymer optics by adopting 3D graphene network coated silicon molds with high thermal conductivity. Nevertheless, the heat transfer mechanism including the interface thermal resistance evolution between 3D graphene network coating and polymer has not been thoroughly revealed. In this study, the interface thermal resistance model was established by simplifying the contact situation between the coating and polymethylmethacrylate (PMMA), and then embedding into the finite element method (FEM) model to study the temperature variations of PMMA in surface rapid heating process. Heating experiments for graphene network were then carried out under different currents to provide the initial heat for heat transfer model. In addition, residual stress of the PMMA lens undergoing the non-uniform thermal history during molding was presented by the simulation model together. Finally, the optimal molding parameters including heating time and pressure will be determined according to calculation results of the interface thermal resistance model and microlens array molding experiment was conducted to illustrate that the interface thermal resistance model can predict the temperature of the polymer to achieve a better filling of microlens array with smooth surface and satisfactory optical performance.

## 1. Introduction

With the rapid development of high-resolution cameras and lighting systems, polymer optical elements with micro-scale three-dimensional structures have gained growing attention for their novel applications. Microlens arrays, due to its control of light reflection, refraction and diffraction, can realize complex imaging, rapid positioning, range and speed measurement, navigation, beam guidance and optical communication in civil and military applications. Given their merits of small volume and mass and high sensitivity, many researchers over the past several decades have contributed huge effort in the fabrication of various microlens array systems. Due to its excellent performance, high-molecular polymers are entering the design domain of advanced optical systems.

Several approaches have been applied to produce microlens array optical components, such as replication technology (including compression molding, hot embossing [1], injection molding [2,3,4], etc.), liquid lens, thermal reflow, DMD maskless lithography, two-photon polymerization 3D printing, etc. [5]. For instance, Chen et al. [6] proposed a vacuum-assisted UV imprinting facility and method for microlens arrays fabrication for artificial compound eye applications. Xu et al. [7] reported a facile method to create microlens arrays with controllable focal length by changing the interfacial energy between the liquid-state acrylate resin and the solidified polydimethylsiloxane (PDMS). Huang et al. [8] combined dose-modulated DMD-based lithography and surface thermal reflow process to achieve high-quality aspheric microlens array fabrication. 

Among the diverse fabrication methods for polymer optical components, precision molding, including compression molding and injection compression molding, is a promising mass-production method. Injection compression molding is famous for its short cycle time and large volume capability. Loaldi et al. [9] used injection compression molding (ICM) to produce enhanced optical performances of molded polymer optics in terms of birefringence and transmission of light. Roeder et al. [10] fabricated a large number of over 12,000 microlenses on surfaces less than 2 cm^2^ by means of ultraprecision milling (UP-milling) and injection compression molding. In addition, they adopted this approach to produce a complicated curved diffractive optical element (DOE) in their another work [11]. However, for injection compression molding, shrinkage is inevitable due to its intrinsic characteristic of processing.

It seems that compression molding enjoys a high reputation in mass production because of its cost-effectiveness and high repeatability, yet it takes a longer processing time [12,13]. The traditional precision molding process includes four steps which are heating, pressing, slow cooling to a medium temperature and rapid cooling to room temperature. In the heating process, the whole polymer preform together with mold assembly is heated up and the polymer turns into the viscoelastic state when the temperature passes the glass transition temperature, *T_g_*. A stable pressure is then exerted on the upper mold to press polymer preform to duplicate the micro-/nano-scale surface topography on its surface. After a slow cooling down, the entire mold assembly restores to room temperature [14,15,16,17]. Under the current traditional route, the heating and cooling process is time-consuming, which is subject to the non-contact heating method and the huge heat capacity of the whole system. Therefore, rapid molding with contact heating was proposed to solve this problem.

Xie et al. [18] proposed an interesting surface heating means in preparation for polymer hot embossing. In this process, a newly-invented low-resistance 3D graphene network on the substrate (e.g., silicon wafer) was employed to produce joule heat when current passing the graphene network. Li et al. [19] introduced this rapid heating means to molding chalcogenide infrared glass at higher temperature. These investigators validated the feasibility and stability of the newly-proposed improved technique in actual molding process. The replication accuracy of the compression system can be up to the micro-meter level. In addition, our previously published work on rapid molding mainly focuses on establishing a simple FEM model to calculate temperature field and refractive index variation of the molded polymer [20]. However, the accompanying issues related to the heat transfer in surface rapid heating process were not fully discussed, such as the contact situation and interface thermal resistance between graphene network coating and the polymer. Since polymer will get soft during the heating and it will be difficult to achieve its temperature, a model that describes the relationship between interface thermal resistance and temperature and pressure is greatly required. This investigation is expected to put forward solutions for these concerns.

In this work, our main contribution is to establish an interface thermal resistance model between coating and polymer and then embed it into an FEM model for surface rapid heating to calculate the temperature distribution of the molded PMMA lens. Firstly, the thermal profiles under different currents—2A, 3A and 4A—were investigated by conducting the current heating experiment. Then, the temperature distribution of the molded polymer can be calculated by the improved FEM simulation with the novel interface thermal resistance model by using these heating profiles as the input temperature. After that, the residual stresses among the entire polymer block under various currents were also achieved to investigate the disequilibrium of internal stress resulted from non-uniform heating. Eventually experiments of microlens array rapid molding were carried out under the calculated heating time and molding pressure to verify that the polymeric microlens array has refined geometrical shape and optical performance, thereby illustrating the interface thermal resistance model is accurate to predict the temperature with knowledge of basic parameters of coating and polymer.

## 2. FEM Simulation with the Interface Thermal Resistance Model

### 2.1. Viscoelastic Model of PMMA at Evaluated Temperatures

Polymers are very sensitive to strain rate at high temperatures and exhibit obvious viscous properties. The deformation process after stress is a process that changes with time, and the recovery process after unloading is a delayed process. Therefore, the stress of this type of material is not only related to the strain at the time, but also related to the entire history of strain. The corresponding relationship between stress and strain will no longer exist. This type of material is called viscoelastic material.

Above the glass transition temperature (*T_g_*), PMMA exhibits significant viscoelasticity, that is, it has the dual characteristics of solid elasticity and fluid viscosity. The stress response of viscoelastic PMMA under constant stress includes three parts: instantaneous elastic deformation, hysteretic elastic deformation and linear viscous deformation. Elastic deformation has the characteristics of transient response. Stress and strain become proportional to Hooke’s law, while viscous flow is related to time, and the deformation relationship obeys Newton’s law of flow. Through the series and parallel combination of elastic elements and viscous elements, many models describing the properties of viscoelastic materials have been established, including Kelvin model, Burgers model and Maxwell model.

To more accurately describe the viscoelastic deformation characteristics of PMMA at high temperatures, multiple Maxwell models are usually combined in parallel, that is, the generalized Maxwell model.

The time-dependent stress–strain response can be described by the following formula:

(1) $$\sigma =\int_{0}^{t}2E\left(t-\tau \right)d\tau$$

In Equation (1), *t* is the current time, *τ* is the time in the past, and *E*(*t* − *τ*) can be described by the Prony series:

(2) $$E\left(t-\tau \right)=E_{0}\left[\alpha_{\infty }+\overset{n_{E}}{\underset{i=1}{\sum }}\alpha_{i}\exp\left(-\frac{t}{\tau_{i}}\right)\;\right]$$

where *τ_i_* is the relaxation time, *α_i_* is the weighting factor, *n_E_* is the number of units in the generalized Maxwell model, and *E*_0_ is the initial modulus of the unit. The weight coefficient and relaxation time satisfy the following two formulas:

(3) $$\overset{n}{\underset{i=1}{\sum }}\alpha_{i}=1$$

(4) $$\tau_{i}=\frac{\eta_{i}}{E_{i}}=\frac{\eta_{i}}{E_{0}\alpha_{i}}$$

where *η_i_* is the viscosity of the damping in each Maxwell unit. The above formulas could completely describe the viscoelasticity of PMMA at high temperature. The viscoelastic characteristics of the material can be measured through experiments [21], and it is a necessity to fit the stress relaxation curve or the creep curve to obtain the relevant parameters. According to the material test data [21], the Prony series is determined by fitting the data analysis software, as shown in Table 1.

The mechanical properties of viscoelastic materials change with temperature. The stress relaxation characteristics at different temperatures can be represented by a simple thermo-rheological model. That is, the stress relaxation characteristic curves at different temperatures can be measured by setting the stress relaxation curve at the reference temperature *T_ref_* on the logarithmic time axis. It can be obtained by moving up without changing the shape of the curve. The amount of translation is defined as *α_T_* (*T*). Similarly, the stress relaxation time *τ* (*T*) at different temperatures can be also calculated by the amount of translation *α_T_* (*T*):

(5) $$\log\tau \left(T\right)=\log\left(\alpha_{T}\right)+\log\tau \left(T_{ref}\right)$$

The William–Landel–Ferry model is selected for describing thermal rheological characteristics modeling of PMMA at high temperature.

(6) $$\log\alpha_{T}=\frac{-C_{1}\left(T-T_{ref}\right)}{C_{2}+\left(T-T_{ref}\right)}$$

The values of the three parameters *T_ref_*, *C*_1_ and *C*_2_ are shown in Table 2.

### 2.2. Modeling of Interface Thermal Resistance between PMMA and Graphene Network

Two cases of thermal conduction conditions in the rapid molding are considered. One is the thermal conduction at the graphene-polymer interface. The 3D graphene coating can generate heat and transfer to the bottom surface of the polymer block, which is a contact heating way compared with infrared heating in conventional compression molding. It can be expressed by the following equation [22]:

(7) $$-k\frac{\partial T}{\partial l}=h_{m}\left(T-T_{m}\right)$$

where *k* means the thermal conductivity. *h_m_* indicates the interface heat transfer coefficient between 3D graphene network and polymer preform, relating to many factors such as interface pressure and mold surface roughness [23]. *T_m_* is the graphene network temperature.

The other case of heat transfer is the thermal conduction inside the polymer block, which can be expressed by Equation (7) [22]:

(8) $$\rho C_{p}\frac{\partial T}{\partial t}=k\nabla^{2}T$$

where *ρ* is the preform density. *C_p_* is specific heat, and *k* is thermal conductivity.

**Figure 1:**
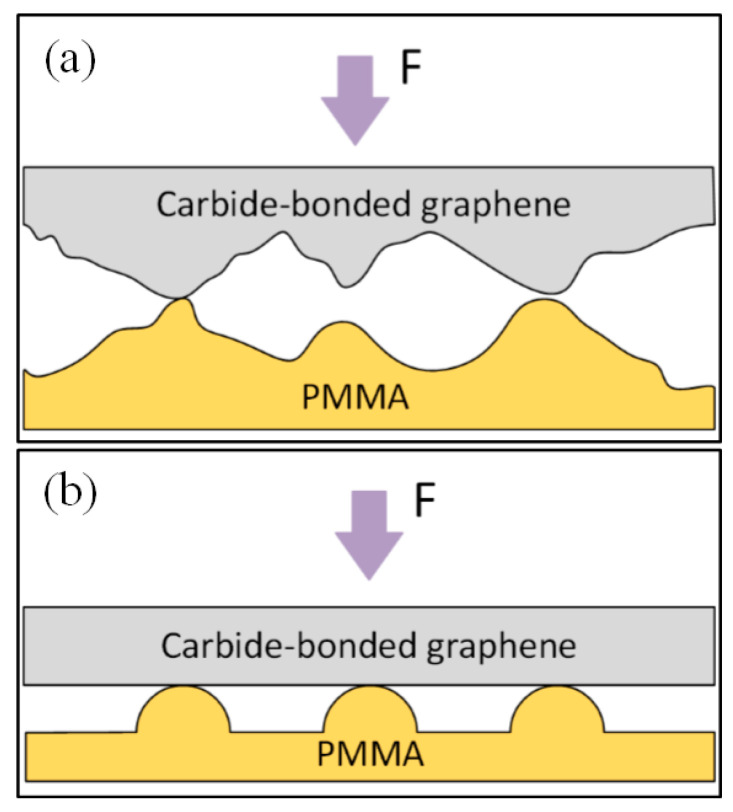
Schematic of the equivalent contact model of the PMMA-graphene interface: (**a**) actual contact between grapheme coating and PMMA; (**b**) equivalent contact between grapheme coating and PMMA.

During the modeling process, the interface heat transfer coefficient in the graphene–PMMA interface heat transfer model is a function that needs to consider the graphene coating and PMMA surface morphology, contact pressure, and thermodynamic properties [24]. According to the Hertz contact theory [25], the actual contact area between the graphene coating and the PMMA interface is composed of a series of continuous protrusions between two contact surfaces, and the heat conduction between the interface mainly depends on the continuous protrusion contact part. As the graphene coating and PMMA preform have similar surface roughness (both are about 10 nm), when calculating the interface contact, it can be equivalent to the contact of two surfaces with the same roughness. To further simplify the calculation, the contact interface can be regarded as micro-contact with a series of periodic micro-protrusion arrays. Based on this assumption, the equivalent contact model of the graphene–PMMA interface is established, as shown in Figure 1.

The interface heat transfer coefficient is the reciprocal of the interface contact thermal resistance. According to the physical definition of thermal resistance, the thermal resistance coefficient between interfaces can be defined as:

(9) $$h_{m}=\frac{1}{\theta }=\frac{k^{*}s}{2r^{*}}$$

(10) $$r^{*}=\sqrt{r_{1}{}^{2}+r_{2}{}^{2}}$$

(11) $$k^{*}=\frac{2k_{1}k_{2}}{k_{1}+k_{2}}$$

where r*, *r*_1_ and *r*_2_ are equivalent surface roughness, PMMA surface roughness and graphene surface roughness, respectively; k*, *k*_1_ and *k*_2_ are equivalent thermal conductivity, thermal conductivity of PMMA and thermal conductivity of graphene, respectively; *s* is the interface contact ratio of PMMA and graphene.

According to the simplified contact model, a single contact radius can be calculated as [25]:

(12) $$R=\frac{4r^{*}}{\pi }$$

The contact pressure in the initial heating stage is very small, hence elastic contact is mainly considered between the contact interfaces. According to the Hertzian contact theory of elastic contact [25], there are the following formulas:

(13) $$a^{3}=\frac{4}{3}\frac{PR}{E^{*}}$$

(14) $$\frac{1}{E^{*}}=\frac{1-\upsilon_{1}{}^{2}}{E_{1}}+\frac{1-\upsilon_{2}{}^{2}}{E_{2}}$$

In the formula, *P* is the contact pressure, *R* is the radius of each micro-protrusion, E* is the equivalent elastic modulus, *a* is the contact area, E*, *E*_1_, and *E*_2_ are the equivalent elastic modulus and the elastic modulus of PMMA, respectively. The elastic modulus of graphene, *υ*_1_ and *υ*_2_ are the Poisson’s ratio of PMMA and graphene, respectively.

In summary, the formula for calculating the contact ratio can be obtained as:

(15) $$s=\frac{2a}{4R}=\frac{1}{2}{\left(\frac{3}{4}\frac{P}{E^{*}R^{2}}\right)}^{\frac{1}{3}}$$

According to the above formula, the interface heat transfer coefficient can be calculated by measuring the roughness of PMMA and graphene, the heat transfer coefficient, the relationship curve of the elastic modulus with temperature, and the mold pressure. It can be seen from Equation (16) that the interface heat transfer coefficient is inversely proportional to the material modulus and roughness, and directly proportional to the contact pressure. The greater the pressure, the greater the heat transfer coefficient and the faster the heat transfer.

(16) $$h_{m}=\frac{k^{*}}{4r^{*}}{\left(\frac{3P}{4E^{*}R^{2}}\right)}^{\frac{1}{3}}$$

**Figure 2:**
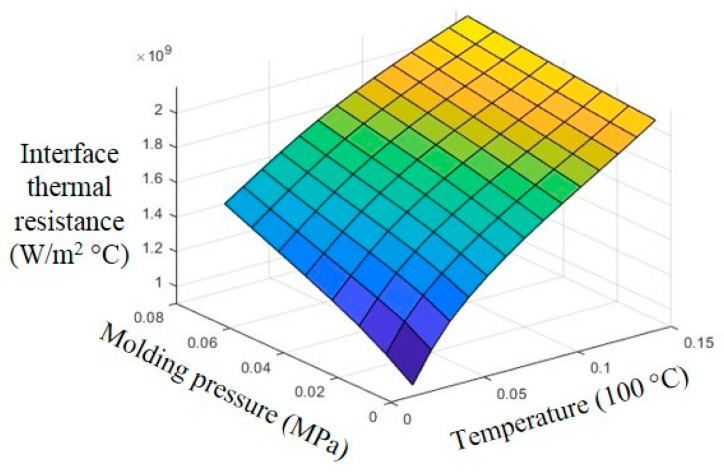
Effects of temperature and pressure on interface thermal resistance.

In the finite element heating simulation, by importing the thermal-mechanical properties of PMMA and graphene into the interface thermal conductivity model, the thermal conductivity of the graphene–PMMA interface at different temperatures and pressures can be obtained, as shown in Figure 2. 

It can be seen from Figure 2 that the thermal conductivity becomes larger as the temperature and pressure increase. Substituting the graphene–PMMA interface thermal conductivity *h_m_* into the heat conduction model, the complete thermal boundary condition of graphene–PMMA in surface rapid heating can be expressed by Equation (17):

(17) $$-k\frac{\partial T}{\partial l}=\frac{k^{*}}{4r^{*}}{\left(\frac{3P}{4E^{*}R^{2}}\right)}^{\frac{1}{3}}\left(T-T_{m}\right)$$

### 2.3. FEM Simulation

**Figure 3:**
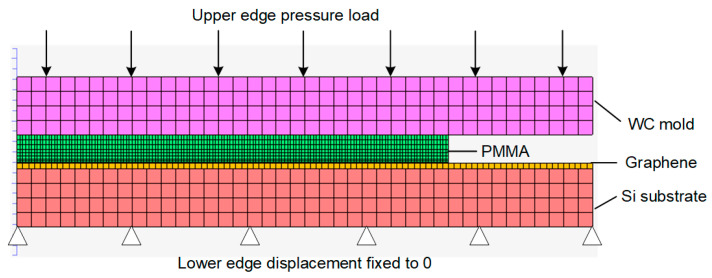
2D FEM model for surface rapid heating.

The surface heating process is hard to investigate by experiment when lacking in in-situ measurements. Hence, a 2D axisymmetric simulation model for graphene heating was constructed within a commercial FEM code MSC MARC [26,27,28,29], as illustrated in Figure 3. This model can be used for description of the temperature and optical properties co-dependency in the polymer molding process.

**Figure 4:**
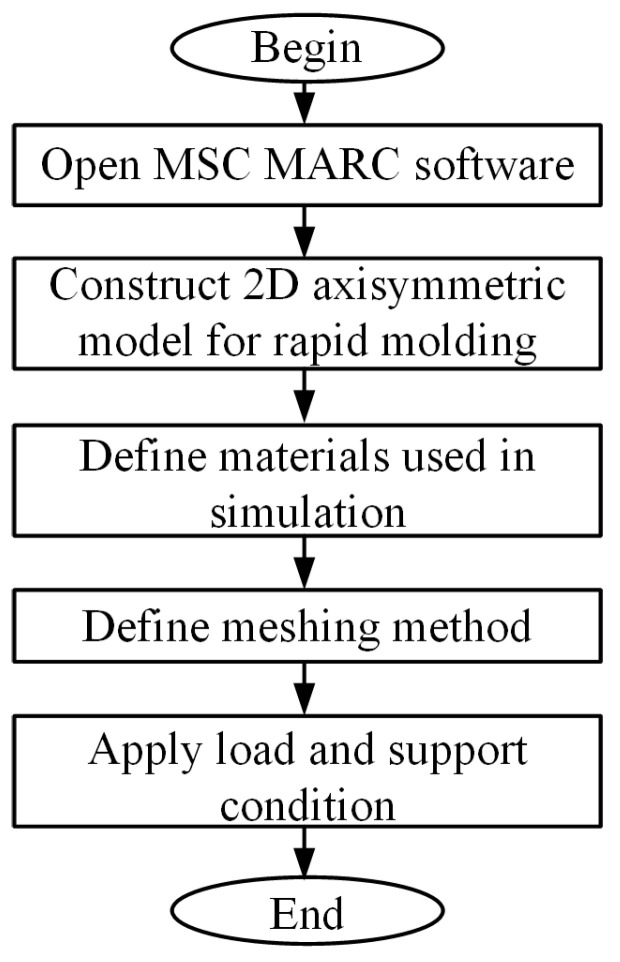
Flow chart of the simulation for surface rapid heating.

As displayed in Figure 3, a PMMA preform was placed between the upper mold and silicon substrate coated with graphene. Due to no structure on the substrate surface, the glass flow along the horizontal direction can be ignored during the molding process. The two-dimensional finite element calculation can achieve the calculation purpose. Therefore, only half of the model was built to avoid consuming unnecessary computing resources. The polymer and two molds were set to be symmetrical on the left and right boundaries. The PMMA was completely free of constraints except its surface contact with both upper mold and silicon substrate. The silicon substrate was fixed on the machine base and the upper mold was moved vertically downward to press the PMMA preform with a constant pressure. In the simulation model, the upper mold, the PMMA preform, and the substrate were set to deformable bodies yet meshed into quadrilateral elements with different sizes based on the requirements for the calculation results. Especially for the PMMA, we focused more on its surface adjacent to graphene, so we refined the grids for that part. The calculation was performed by exerting different heating profiles on the graphene network until the PMMA block arrived at the temperature of 150 °C and then cooled to room temperature. The flow chart of the simulation is presented in Figure 4.

## 3. Experiments

### 3.1. 3D Graphene Network for Polymer Hot Embossing

Graphene has been strongly attracting researchers’ attention since it was discovered in 2004 [30]. As a type of two-dimensional (2D) material, graphene has weak adhesion that cannot be adopted as strong coating due to weak van der Waals force among its atomic layers. To solve this problem, Huang et al. [31], prepared a new 3D graphene network by using chemical vapor deposition (CVD). Apart from high stability and durability, the new graphene network coating can be treated as high-efficiency heating film thanks to its high electrical conductivity at 1.98 × 10^4^ S/m and low resistivity of 20.4 mΩ [18]. In this study, the 3D graphene network was deposited on both plane silicon surface and micro dimple array silicon mold surface for current heating and microlens array rapid molding experiments, respectively.

### 3.2. Heating Experiments for PMMA

**Figure 5:**
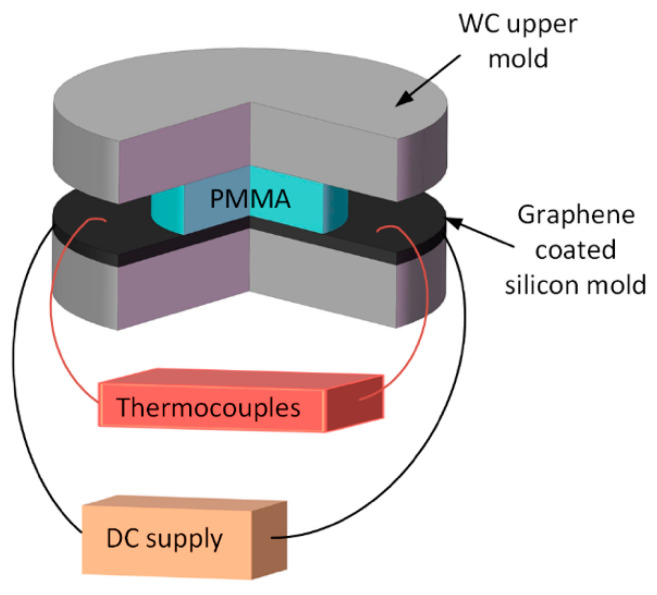
Surface rapid heating assembly.

To achieve the heating profiles of the graphene network, a current heating experiment was performed. Since we need to obtain the true temperature increase of graphene coating in polymer molding process, a plane graphene-coated silicon substrate and a PMMA block were adopted to investigate the heating and cooling profiles of the graphene coating with the PMMA being placed on its surface under a certain molding force. As shown in Figure 5, a graphene-coated silicon wafer and a PMMA preform (CM205, CHIMEI, Tainan, Taiwan) were successively assembled between the tungsten carbide upper and lower molds. A data acquisition system was introduced to monitor temperature variation of the graphene coating by K type thermocouple (T40-P7-30-SF0-1, Fluke Corporation, Everett, WA, US) with accuracy of 0.1 °C. Two copper electrodes for the purpose of electric conduction were fastened on both sides of the coating surface and a TDK-Lambda Programmable DC power Supply (Z^+^100-2, TDK-Lambda, Tokyo, Japan) was employed to provide current through copper electrodes for the graphene coating. A servomotor (L-412, Physik Instrumente GmbH, Karsruhe, Germany) provided a constant pressure on the upper mold together with position closed-loop control of the mold. The precision can be up to 0.6 μm. The loading of 40N was applied in the overall process, including the unheated stage and the heating stage, and the force senor has an accuracy of 1N.

## 4. Results and Discussion

### 4.1. Temperature Distribution under Various Currents

**Figure 6:**
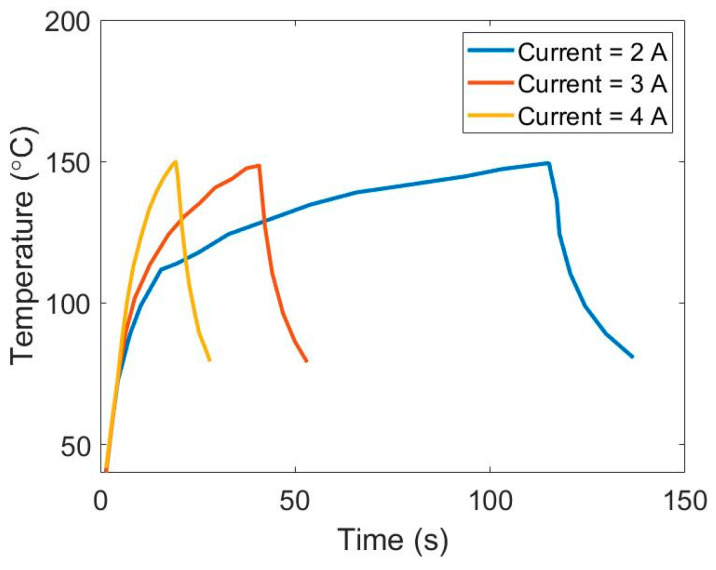
Heating/cooling response of graphene network under various currents of 2A, 3A and 4A.

In the heating experiments, various currents were applied to the graphene network coated mold until the temperature was 150 °C and then cut off to cool down to around 80 °C. Figure 6 shows the heating and cooling profiles of the 3D graphene network under currents of 2A, 3A and 4A. It can be seen from Figure 6 that a larger current leads to a larger temperature increase, yet smaller growth rate, which may be caused by resistance variation under different currents. In addition, we should notice that when current equals to 2A, the temperature curve exists a turning point at temperature of about 110 °C where its growth rate suddenly dropped. By contrast, when currents are 3A and 4A, there were no turning points and the growth rates under two currents both gradually got smaller. For cooling response, the condition with 2A current requires more time compared with the two other conditions.

**Figure 7:**
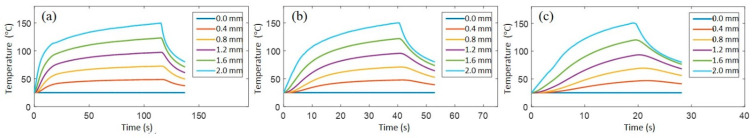
Temperature variation vs. time at the interior nodes at different depths under different currents: (**a**) 2A; (**b**) 3A; (**c**) 4A.

After obtaining the temperature curves with time under different currents, we input it in the FEM simulation to calculate the temperature among the PMMA block. Figure 7a–c shows the temperature variation vs. time at the interior nodes at different depths when the bottom of the PMMA block reaches 150 °C under currents of 2A, 3A and 4A. During surface rapid heating, heat is flowing from the graphene coating to the bottom surface of the polymer block and slowly conducted within the PMMA body. Subject to the low thermal conductivity of the polymer material, the bulk temperature of PMMA never reached *T_g_* (~110 °C) in one-time heating and cooling cycle, which is truly energy-saving and efficient. We can conclude from Figure 7 that for a relatively small current such as 2A, the temperature of the bottom of the polymer block where adjacent to the coating increases dramatically at the beginning reaching 110 °C in the first 20 s and then slows down to a ramp until it reaches the molding temperature (150 °C) with a total heating time of approximately 120 s. By contrast, when the current is 4A, temperature rises quickly straight to the molding temperature with only an imperceptible speed down and the entire process ends in less than 20 s. By using the FEM simulation model, we can predict the temperature distribution in any location of the entire polymer block.

### 4.2. Residual Stress Distribution of PMMA

**Figure 8:**
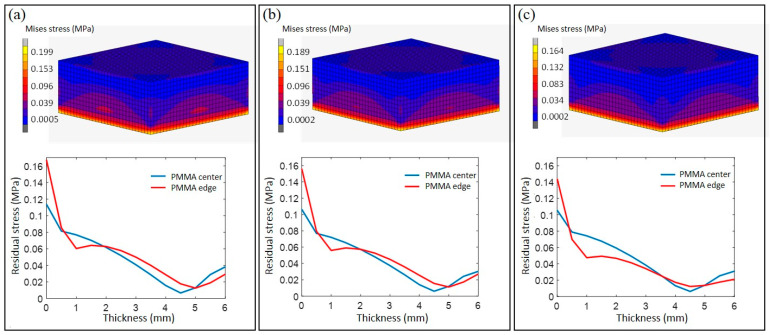
Residual stress distribution varies with thickness under different currents: (**a**) 2A; (**b**) 3A; (**c**) 4 A.

During a rapid molding process, part of PMMA changes from a solid state to a viscoelastic state and then back to a solid state, so that the residual stress mostly exists in the region of the molded optical element which is adjacent to the graphene coating. Given that the residual stress is mainly caused by the uneven distribution of the temperature field during the heating and cooling process, the residual stress in the rapid molding tends to be more severe. Therefore, we need to investigate the residual stress distribution and limit it in a specific level. When the temperature is still higher than *T_g_*, the temperature gradient in PMMA is still very small. As the material viscosity is low at this time, and the generated stress can be released in a short time, the stress is still very small at this time. When PMMA continues to cool below *T_g_*, especially when the surface temperature of PMMA has cooled to room temperature, and the center needs further cooling, due to the existence of the coefficient of thermal expansion, the center of PMMA begins to shrink. At this time, compared to the surface of PMMA, the center appears as tensile stress, and the resulting stress will be enclosed in PMMA. Residual stress is an important indicator for the evaluation of optical components. Excessive residual stress during the molding process can lead to refractive index changes, wavefront distortion and imaging distortion. By extracting the Mise stress from the calculation results of the surface rapid molding model, the change rule of the residual stress distributions in the PMMA under currents of 2A, 3A and 4A were obtained, as shown in Figure 8.

Figure 8 shows that the residual stress of PMMA after surface rapid molding under power-on conditions is mainly concentrated in the part of the thermally deformed layer, while the residual stress in the area outside the thermally deformed layer is very small. The residual stress vs. thickness distribution curve can more intuitively show this in conclusion. At the same time, as the voltage increases, the residual stress distribution area becomes smaller, and the maximum value becomes smaller. When the currents are set as 2A, 3A and 4A, the maximum value of residual stress is 0.199 MPa, 0.189 MPa and 0.164 MPa, respectively. The higher the voltage, the shorter the heating time, the smaller the thermally deformed layer, and the smaller the residual stress value. Similarly, the inconsistency of the internal and external cooling rates of PMMA causes the residual stress at the edges to be slightly larger than the residual stress at the center, which is also reflected in the curve.

### 4.3. Fabrication of Polymeric Microlens Array and Its Optical Tests

**Figure 9:**
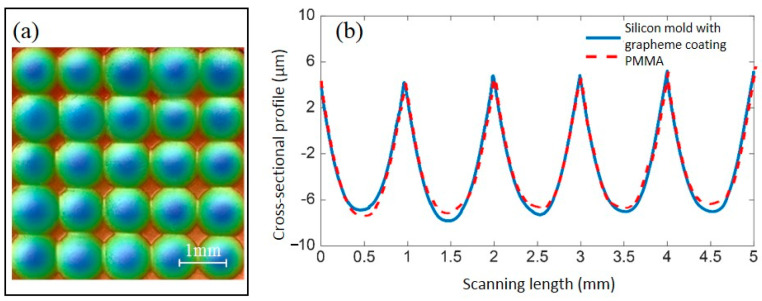
Measurement of the PMMA microlens array: (**a**) measured 5 × 5 microlens array on PMMA block; (**b**) the profile comparison between graphene-coated silicon mold and PMMA.

Since the temperature distribution of polymer could be accurately calculated by the FEM simulation with the interface thermal resistance model embedded in, the relationship between heating time and temperature of the PMMA block was achieved. Thus, a protocol for PMMA microlens array rapid hot embossing was proposed, including the optimal time for PMMA reaching the molding temperature under various currents and molding forces. To validate the simulation, a rapid hot embossing experiment for PMMA microlens array was conducted. The silicon substrate with 5 × 5 micro dimple array was machined and then coated with the graphene network. After that, a conjugated microlens array pattern was formed on the PMMA surface, as shown in Figure 9a. Figure 9a plots the geometrical shape of the matrix of microlens array by adopting a non-contact profilometer (Wyko NT 9100, Warsaw, NY, USA) with an accuracy of 1 nm. Figure 9 shows that the uniformity of microlenses is fine with very close height and aperture, although the shape of each lens is not in a perfect circle due to the defective silicon mold. To eliminate the effect of the imperfect mold, we detected the filling of the PMMA to evaluate the hot embossing process. A line scan was employed to inspect the detail replication condition and Figure 9b presents the profile comparison between the mold and polymer of five individual microlenses. It can be seen from the comparison that the geometrical shape of the polymeric microlenses is accurately determined by the micro dimple mold with a filling rate of approximately 95% in height, resulting in perfect replication of the microlens array pattern on the PMMA block. We can conclude that the interface thermal resistance model can be embedded in the FEM simulation to precisely predict the heating time for graphene network and create microlens array in predesigned shape.

**Figure 10:**
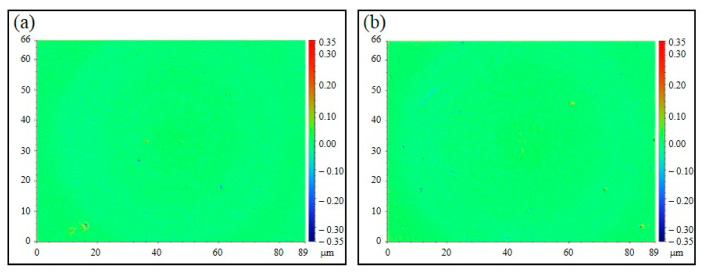
Surface roughness comparison: (**a**) micro dimple on the graphene network surface; (**b**) microlens on the PMMA surface.

In addition to shape accuracy, surface roughness is also one of the most significant indices to evaluate the functionality of an optical element. The true surface roughness of each microlens was measured by the profilometer with no tilt or sphere because of the curved microlens profile. The surface roughness of the graphene-coated mold and the molded polymer were measured as 7.8 nm and 9.2 nm, respectively, as shown in Figure 10a,b. The result shows that the Ra value evolution from the mold surface to the polymer surface has not experienced much change, indicating that the elaborate rapid molding process with accurate heating time and pressure achieves the ideal replication of surface roughness as well.

**Figure 11:**
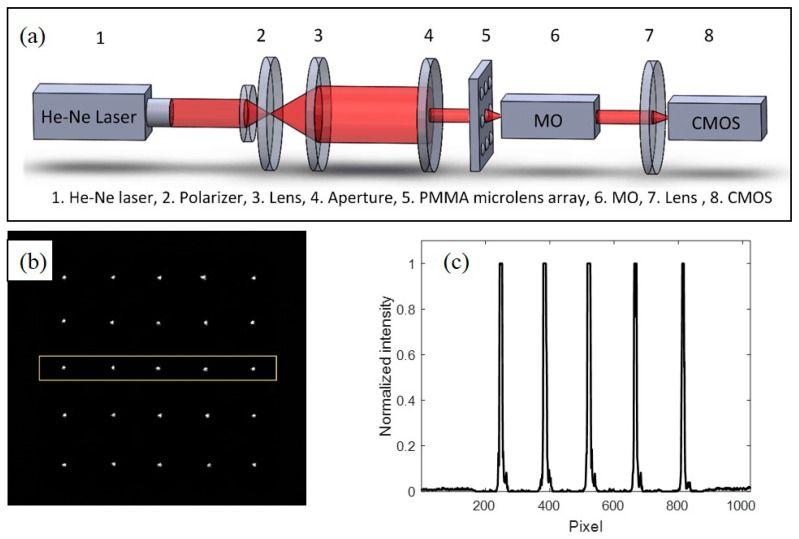
Optical measurement: (**a**) schematic of the setup for detecting the optical performance of the microlenses; (**b**) focal point picture captured by CMOS; (**c**) intensity data of five adjacent focal points.

As aforementioned, residual stress will harm the optical function of the microlens array due to the uneven quick heating and cooling of the polymer. Therefore, accurate geometrical data is not adequate to enable the optical element function well unless its optical performance is verified. In this study, an optical measurement setup was built as presented in Figure 11a to test the dimension of the focal points and the focal length of the tested microlens array. In this setup, light source was provided by He-Ne laser and the laser beam was spatially filtered and then expanded to a collimated light. According to the size of each microlens, an aperture was set to adjust the diameter of the light beam. When the laser passed through the microlens, it could be collected by a lens imaging system and the focal spot image of the microlens array can be observed by a CMOS camera. Figure 11b,c illustrate a photograph of the focal spots and the intensity distribution for five adjacent spots in the middle line. It can be seen that the spots were uniformly spaced and the intensity for each spot was also uniform, and that the spot possessed a uniform intensity, indicating that the molded microlens array has qualified optical performance.

## 5. Conclusions

In this paper, surface rapid heating process using newly-developed 3D graphene-based network is an economic and low-cost fabrication method for polymeric optical components with high efficiency. Several conclusions are drawn:

(1) The interface thermal resistance evolution between 3D graphene network and polymer has been thoroughly investigated based on Hertz contact theory that the relationship between interface thermal resistance and molding pressure and temperature was achieved.
(2) By applying this model into the FEM simulation, the temperature variation of the entire polymer block was predicted. In addition, the FEM simulation with the interface thermal resistance model can be used to calculate the residual stress of the molded polymer.
(3) Since the temperature and residual stress distribution of polymer have been predicted by the calculation, a microlens array surface rapid molding was conducted to demonstrate that the polymeric microlens array optical component with delicate geometrical shape, good surface roughness and feasible optical performance can be achieved by setting parameters according to the simulated results.

By adopting the interface thermal resistance model, the surface rapid heating strategy can provide a large-production optical fabrication approach with high quality and much shorter cycle time.

## Figures and Tables

**Table 1:** The parameters of Prony series fitting for PMMA viscoelastic model.

*α_i_*	*τ_i_*
7.92 × 10^−5^	0.2106
0.00612	0.3385
0.179	0.2573
2.67	0.1362
27.5	0.0396
216	0.0128
2490	0.0032
48,900	0.001
1.94 × 10^6^	0.0004
1.39 × 10^8^	0.00015

**Table 2:** The parameters for the William–Landel–Ferry model.

Reference Temperature *T_ref_* (°C)	*C*_1_	*C*_2_
109	16	56

## Data Availability

The data presented in this study is openly available.
